# Improved Parameter Identification for Lithium-Ion Batteries Based on Complex-Order Beetle Swarm Optimization Algorithm

**DOI:** 10.3390/mi14020413

**Published:** 2023-02-09

**Authors:** Xiaohua Zhang, Haolin Li, Wenfeng Zhang, António M. Lopes, Xiaobo Wu, Liping Chen

**Affiliations:** 1 College of Automation, Zhongkai University of Agriculture and Engineering, Guangzhou 510225, China; 2 Guangdong-Hong Kong-Macao Greater Bay Area Agricultural Products Digital Logistics Research Center, Guangzhou 510225, China; 3 College of School of Mechanical and Electrical Engineering, Zhongkai University of Agriculture and Engineering, Guangzhou 510225, China; 4 Guangdong Agricultural Products Cold Chain Transportation and Logistics Engineering Technology Research Center, Guangzhou 510225, China; 5 LAETA/INEGI, Faculty of Engineering, University of Porto, Rua Dr. Roberto Frias, 4200-465 Porto, Portugal; 6 School of Electrical Engineering and Automation, Hefei University of Technology, Hefei 230009, China

**Keywords:** FO equivalent circuit, parameter identification, beetle swarm optimization

## Abstract

With the aim of increasing the model accuracy of lithium-ion batteries (LIBs), this paper presents a complex-order beetle swarm optimization (CBSO) method, which employs complex-order (CO) operator concepts and mutation into the traditional beetle swarm optimization (BSO). Firstly, a fractional-order equivalent circuit model of LIBs is established based on electrochemical impedance spectroscopy (EIS). Secondly, the CBSO is used for model parameters’ identification, and the model accuracy is verified by simulation experiments. The root-mean-square error (RMSE) and maximum absolute error (MAE) optimization metrics show that the model accuracy with CBSO is superior when compared with the fractional-order BSO.

## 1. Introduction

Electric vehicles (EVs) are proving to be a viable alternative to internal combustion engine-powered vehicles [1,2]. Among all types of fuel cells, the LIB is one of the most promising energy sources, because of its high energy capacity and Coulombic performance [3,4,5,6,7], with great potential in EV applications. LIBs are a complex nonlinear time-varying system with multiple real-time changing quantities [8,9,10,11,12,13], including state of charge, state of health, state of power, and state of energy. State estimations are all dependent on accurate battery models. In general, a good battery model should accurately describe the static and dynamic battery behavior; involve low computational resources; and be relatively simple to implement in battery management systems [14]. However, a simple and precise battery model is not readily available. Commonly used battery models include electrochemical models [15], neural networks [16], thermal models [17], and equivalent circuit models (ECMs) [18]. ECMs are made of capacitances and resistances and are mainstream due to their low computational burden. Moreover, fractional-order ECMs have attracted growing attention in the arena of battery modeling, demonstrating better fitting capability relative to their integer-order counterparts [19,20].

Fractional-order models (FOMs) have many parameters that cannot be directly measured, such as fractional order [21,22]. Regardless of whether the model parameter values are regarded as constant or time-varying, parameter identification for the original model is necessary for equivalent models of batteries [23]. Parameter identification methods, required for the characterization of LIBs, have been widely investigated. For example, the least square method [24], particle swarm optimization (PSO) [25], computational intelligence techniques [26], and genetic algorithms [27] use the statistical characteristics of the battery transient behaviors as the principle of optimization to find the most suitable battery parameters that can fit the battery transient characteristics well.

The BSO algorithm is a new meta-heuristic algorithm, and its effectiveness and superiority have been verified [28]. Embedding the CO operators concept in the core of the optimization algorithms leads to superior performance during the search process. This is due to the fact that the particles’ memory is captured by the CO derivative [29]. For another, mutation operations prevent the algorithm from entering local minima. In view of the advantages of both, the above two methods are applied to BSO to obtain CBSO. Furthermore, the proposed CBSO is employed to identify the model parameters of LIBs, and the numerical experiments show that the CBSO can improve model accuracy.

The paper is organized as follows: The preliminary concepts about fractional calculus are introduced in Section 2. Section 3 provides details about the mathematical deduction of the FOM for LIBs. In Section 4, combined with the experimental results, the algorithm performances are discussed, and comparisons with other algorithms are included. Finally, Section 5 concludes the paper.

## 2. Preliminaries

Fractional-order (FO) differential and integral are expressed as follows, where *q* represents the fractional order (non-integers):

(1) $$\begin{array}{c} D^{q}x\left(t\right)=\left\{\begin{array}{c} \frac{d^{q}}{dt^{q}}x\left(t\right),q>0\\ x(t),q=0\\ \frac{d^{q}}{dt^{q}}=I^{q}x\left(t\right),q<0 \end{array}\right. \end{array}$$

There are mainly three definitions of fractional-order calculus (FOC): the Grünwald–Letnikov (GL), Riemann–Liouville (RL), and Caputo definitions [30]. Generally speaking, the RL definition is commonly used in theoretical analysis research. The Caputo derivative is suitable for the description and discussion of initial value problems of fractional order. The GL definition is suitable for problems that require discretization. Herein, the GL definition is adopted due to its straightforward computational implementation. The GL operator is given by

$$\begin{array}{ccc} D^{q}x\left(t\right) & = & \lim_{T\to 0}\frac{1}{T^{q}}\times \sum_{j=0}^{\left[t/T\right]}{\left(-1\right)}^{j}\langle q,j\rangle x\left(t-jT\right) \end{array}$$

for (n−1<q<n), and 〈q,j〉 stands for Newton binomial coefficient with the following definition:

(2) $$\langle q,j\rangle =\frac{\Gamma (q+1)}{\Gamma (j+1)\Gamma (q-j+1)}$$

where Γ() represents the Gamma function.

We can obtain the two following operators by grouping the conjugate order derivatives [29]:

(3) $$\begin{array}{ccc} \varphi_{1}\left(\alpha ,\beta \right)\left(f\right) & = & \frac{D^{\alpha +j\beta }+D^{\alpha -j\beta }}{2}\left(f\right)\\ \varphi_{2}\left(\alpha ,\beta \right)\left(f\right) & = & \frac{D^{\alpha +j\beta }-D^{\alpha -j\beta }}{2j}\left(f\right), \end{array}$$

where f∈R is a function. Using the Taylor expansions of order r in the neighborhood of *z* = 0, we can obtain

(4) $$\begin{array}{ccc} \varphi_{1}\left(\alpha ,\beta \right) & = & 1-\alpha z^{-1}+\frac{1}{2}\left(\alpha^{2}-\alpha -\beta^{2}\right)z^{-2}\\ & - & \frac{1}{6}\left[\alpha^{3}-3\alpha^{2}+\alpha \left(2-3\beta^{2}\right)+3\beta^{2}\right]z^{-3}+\ldots \\ \varphi_{2}\left(\alpha ,\beta \right) & = & -\beta z^{-1}-\frac{1}{2}\beta \left(1-2\alpha \right)z^{-2}\\ & - & \frac{1}{6}\beta \left[3\alpha^{2}-6\alpha -\beta^{2}+2\right]z^{-3}-\ldots , \end{array}$$

In addition, we can define the operators $\psi_{1}\left(z^{-1}\right)$ and $\psi_{2}\left(z^{-1}\right)$ such that

(5) $$\begin{array}{ccc} \psi_{1}\left(\alpha ,\beta \right) & = & \frac{1}{T^{\alpha }}\{\varphi_{1}\left(z^{-1}\right)cos\left[\beta ln\left(T\right)\right]\\ & + & \varphi_{2}\left(z^{-1}\right)sin\left[\beta ln\left(T\right)\right]\}\\ \psi_{2}\left(\alpha ,\beta \right) & = & \frac{1}{T^{\alpha }}\{\varphi_{2}\left(z^{-1}\right)cos\left[\beta ln\left(T\right)\right]\\ & - & \varphi_{1}\left(z^{-1}\right)sin\left[\beta ln\left(T\right)\right]\}, \end{array}$$

If the sampling time *T* is equal to one, then we can obtain $\psi_{1}\left(z^{-1}\right)=\varphi_{1}\left(z^{-1}\right)$ and $\psi_{2}\left(z^{-1}\right)=\varphi_{2}\left(z^{-1}\right)$.

## 3. Fractional-Order Modeling of LIBs

**Figure 1:**
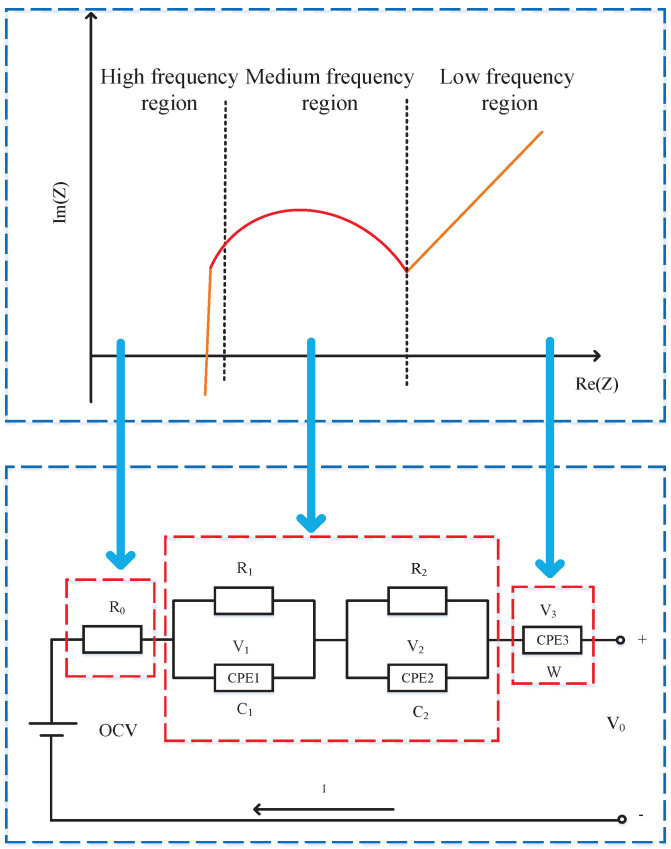
The EIS-FOM representation of LIBs.

The most popular battery model is ECMs, which utilize the ideal resistors, capacitors, and voltage sources to describe battery characteristics. The ECMs include integer-order ECMs and fractional-order ECMs. The FOM uses fractional impedance elements (e.g., constant-phase element (CPE) and Wahlberg element) to describe the electrochemical processes such as charge transfer reaction, electron layer effects, mass transfer, and diffusion of LIBs with sufficiently high accuracy. The phase shift of a FO capacitor is called a phasance [31], and it is an important characteristic parameter of the Nyquist plot in Figure 1. As illustrated, it consists of three sections: (1) a straight line with a constant slope at low frequencies, (2) a semicircle at medium frequencies, and (3) an inductive tail at high frequencies. In particular, the phasance of a Wahlberg element represents the slope of the low-frequency straight line, while for a resistor–capacitor (RC) network, it is related to the shape of the medium-frequency semicircle. Moreover, an ideal Warburg element has a fractional order of 0.5, which produces a straight line with a $45^{\circ }$ slope in the Nyquist plot. Therefore, the differentiation order of the Wahlberg element is fixed at 0.5 in our work.

The typical second-order RC models employ a parallel RC circuit to simulate the impedance spectra of the medium-frequency region. However, ideal capacitors cannot accurately simulate the double-layer effect. Moreover, the low-frequency region of the impedance spectrum is not represented by any electronic components [32]. Therefore, based on the electrochemical process described by the EIS of LIBs, FO impedance elements are introduced to improve model accuracy. Based on the above discussion, a FOM is shown in Figure 1. In the model, the OCV represents the open circuit voltage, I represents the discharge current, $V_{0}$ represents the terminal voltage, $R_{0}$ represents the ohmic internal resistance, $C_{1},C_{2}$, and W are the CPE coefficients, $R_{1}$ and $R_{2}$ are the resistances on the two RC branches, $V_{1}$ and $V_{2}$ represent, respectively, the voltages on the two RC branches, and $V_{3}$ are the Warburg-like element voltage.

The corresponding battery model formulation can be established based on Kirchhoff’s current and voltage laws. The governing equations of the model can be given by

(6) $$\begin{array}{c} \left\{\begin{array}{cc} D^{\alpha_{1}}x_{1}\left(t\right) & =-\frac{V_{1}\left(t\right)}{R_{1}C_{1}}+\frac{1}{C_{1}}u\left(t\right),\\ D^{\alpha_{2}}x_{2}\left(t\right) & =-\frac{V_{2}\left(t\right)}{R_{2}C_{2}}+\frac{1}{C_{2}}u\left(t\right),\\ D^{0.5}x_{3}\left(t\right) & =\frac{1}{W}u\left(t\right),\\ D^{1}x_{4}\left(t\right) & =-\frac{\eta }{Q_{n}}u\left(t\right),\\ y(t) & =OCV\left(x_{4}\left(t\right)\right)-x_{1}\left(t\right)-x_{2}\left(t\right)-x_{3}\left(t\right)-R_{0}u\left(t\right). \end{array}\right. \end{array}$$

where u(t) is the input current; the state vector is defined as x(t) = ${\left[V_{1}\left(t\right),V_{2}\left(t\right),V_{3}\left(t\right),SOC\left(t\right)\right]}^{T}$; and *y*(*t*) represents the output voltage. Moreover, $Q_{n}$ denotes the battery nominal capacity with the unit of ampere-hour (Ah), and η is the Coulomb efficiency. A fourth-order polynomial is used to characterize this nonlinear relationship between *OCV* and *SOC*.

(7) $$\begin{array}{c} OCV=a_{0}+a_{1}SOC+a_{2}SOC^{2}+a_{3}SOC^{3}+a_{4}SOC^{4}, \end{array}$$

where $a_{k}$ (*k* = 0, 1, ⋯, 4) are the coefficients of the polynomial.

The symbol $Q_{n}$ can be expressed as follows:

(8) $$\begin{array}{c} Q_{n}=3600\cdot Q_{AH}\cdot C_{1}\cdot T_{1}, \end{array}$$

where $Q_{AH}$ represents the standard capacity of the battery, $C_{1}$ is the correction factor of the battery cycle life, and $T_{1}$ stands for the correction factor of the battery temperature. In this paper, we do not consider the impact of the battery cycle life or the temperature on the battery capacity. Thus, we set $C_{1}$ = $T_{1}$ = 1.

Generally, a FO system can be expressed as follows:

(9) $$\begin{array}{c} \left\{\begin{array}{ccc} D^{\alpha }x\left(t\right) & = & Ax(t)+Bu(t),\\ y(t) & = & Cx(t)+Du(t). \end{array}\right. \end{array}$$

where the *A*, *B*, *C*, and *D* are the coefficient matrices, and $\alpha =[\alpha_{1},\alpha_{2},\ldots ,\alpha_{n}]$ is the differentiation-order vector. Meanwhile, the system (9) can be discretized in the time based on the GL definition, for k≥1

(10) $$\begin{array}{ccc} x(k+1) & = & [T^{\alpha }A+diag\left(\alpha \right)I]x\left(k\right)\\ & - & \sum_{j=2}^{L+1}{\left(-1\right)}^{j}\frac{\Gamma (\alpha +1)}{\Gamma (j+1)\Gamma (\alpha -j+1)}x\left(k+1-j\right)+T^{\alpha }Bu\left(k\right). \end{array}$$

and for *k* = 0

(11) $$\begin{array}{c} x\left(k+1\right)=\left[T^{\alpha }A+diag\left(\alpha \right)\right]x\left(k\right)+T^{\alpha }Bu\left(k\right). \end{array}$$

Based on Equations (10) and (11), the FO system (6) in its discrete-time representation at the time step *k* can be expressed in the following form (k≥1):

(12) $$\begin{array}{c} \left\{\begin{array}{cc} x(k+1) & =[{\left(\Delta T\right)}^{\alpha }A+diag\left(\alpha \right)I]x\left(k\right)\\ & -\sum_{j=2}^{L+1}{\left(-1\right)}^{j}\left(\begin{array}{c} \alpha \\ j \end{array}\right)x\left(k+1-j\right)\\ & +{\left(\Delta T\right)}^{\alpha }Bu\left(k\right),\\ y(k) & =OCV\left(x_{4}\left(k\right)\right)-x_{1}\left(k\right)-x_{2}\left(k\right)\\ & -x_{3}\left(k\right)-R_{0}u\left(k\right). \end{array}\right. \end{array}$$

where

(13) $$\begin{array}{c} {\left(\Delta T\right)}^{\alpha }=diag\left({\left(\Delta T\right)}^{\alpha_{1}},{\left(\Delta T\right)}^{\alpha_{2}},{\left(\Delta T\right)}^{0.5},\Delta T\right), \end{array}$$

(14) $$\left(\begin{array}{c} \alpha \\ j \end{array}\right)=diag\left(\left(\begin{array}{c} \alpha_{1}\\ j \end{array}\right),\left(\begin{array}{c} \alpha_{2}\\ j \end{array}\right),\left(\begin{array}{c} 0.5\\ j \end{array}\right),\left(\begin{array}{c} 1\\ j \end{array}\right)\right),$$

(15) $$A=\left[\begin{array}{cccc} -\frac{1}{R_{1}C_{1}} & 0 & 0 & 0\\ 0 & -\frac{1}{R_{2}C_{2}} & 0 & 0\\ 0 & 0 & 0 & 0\\ 0 & 0 & 0 & 0 \end{array}\right],B=\left[\begin{array}{c} \frac{1}{C_{1}}\\ \frac{1}{C_{2}}\\ \frac{1}{W}\\ -\frac{\eta }{Q_{n}} \end{array}\right].$$

## 4. Model Parameter Identification and Validation

After the battery model is established, the model parameters need to be identified. An accurate model is the guarantee of battery state estimation. In this section, a novel CBSO algorithm is used for model parameter identification, and the model accuracy is verified by simulation experiments.

Meta-heuristic algorithms have become very popular in model parameter identification because of their stability and flexibility and their ability to better avoid local optimization. The BSO is a new meta-heuristic algorithm and can enhance the performance of swarm optimization through beetle-foraging principles. The BSO is an extension of the beetle antennae search (BAS) algorithm and PSO, and its search efficiency has significant advantages over PSO. This is due to an incremental factor produced by the beetle search, which allows for an effective local search. Therefore, the BSO can alleviate the issues of poor stability and the algorithm falling into local optima [33]. However, it uses little historical information and few measurements. To cope with this problem, the CO operator concept given in Equations (3)–(5) is applied to the BSO. The CBSO embeds this concept in the velocity adaptation laws, and the CO velocity update formulas are as follows:

(16) $$\begin{array}{ccc} \varphi_{1}\left(\alpha_{v},\beta_{v}\right)\left(v_{i}^{k+1}\right) & = & R_{1}\left(P_{best_{i}}^{k}-x_{i}^{k}\right)+R_{2}\left(G_{best_{i}}^{k}-x_{i}^{k}\right),\\ x_{i}^{k+1} & = & x_{i}^{k}+\lambda v_{i}^{k}+\left(1-\lambda \right)\xi_{i}^{k}. \end{array}$$

where $P_{best_{i}}^{k}$ is its best position found so far, $G_{best_{i}}^{k}$ is the best position of the swarm, λ is the proportional constant, and $\xi_{i}^{k}$ denotes the increment factor.

Then, using the CO operators concept given in Equations (3)–(5), one has

(17) $$\begin{array}{ccc} v_{i}^{k+1} & = & \alpha_{v}v_{i}^{k}-\frac{1}{2}\left(\alpha_{v}^{2}-\alpha_{v}-\beta_{v}^{2}\right)v_{i}^{k-1}\\ & + & \frac{1}{6}\left[\alpha_{v}^{3}-3\alpha_{v}^{2}+\alpha_{v}\left(2-3\beta_{v}^{2}\right)+3\beta_{v}^{2}\right]v_{i}^{k-2}\\ & - & \frac{1}{24}[\alpha_{v}^{4}+\beta_{v}^{4}-6\left(\alpha_{v}^{3}-3\alpha_{v}\beta_{v}^{2}\right)\\ & + & 11\left(\alpha_{v}^{2}-\beta_{v}^{2}\right)-6\alpha_{v}]v_{i}^{k-3}\\ & + & c_{1}\left(P_{best_{i}}^{k}-x_{i}^{k}\right)+c_{2}\left(G_{best_{i}}^{k}-x_{i}^{k}\right),\\ x_{i}^{k+1} & = & x_{i}^{k}+\lambda v_{i}^{k}+\left(1-\lambda \right)\xi_{i}^{k}, \end{array}$$

where the velocity update formula uses historical information on velocity based on the CO operator concept. Therefore, it is able to capture the past memory of the particles’ velocity. The updating of the position in each iterative process relies on the individual historical optimal solution and on the optimal global solution of the BSO algorithm. The $\xi_{i}^{k}$ is updated by

(18) $$\begin{array}{ccc} \xi_{i}^{k+1} & = & \delta^{k}\nu_{i}^{k}\cdot sign\left(f\left(x_{ir}^{k}\right)-f\left(x_{il}^{k}\right)\right),\\ \delta^{k+1} & = & \varepsilon \cdot \delta^{k}. \end{array}$$

where $\delta^{k}$ denotes the step size of the *k*-th iteration, *sign*(·) represents a signum function, ε is equal to 0.95, and $f\left(x_{ir}^{k}\right)-f\left(x_{il}^{k}\right)$ represent the scent intensity difference between the right antennae position $x_{ir}^{k}$ and the left antennae position $x_{il}^{k}$ of beetle *i*. The search behaviors of the right antenna and the left antenna are, respectively, expressed as follows:

(19) $$\begin{array}{c} \left\{\begin{array}{c} x_{ir}^{k+1}=x_{i}^{k}+v_{i}^{k}\cdot \frac{d^{k}}{2},\\ x_{il}^{k+1}=x_{i}^{k}-v_{i}^{k}\cdot \frac{d^{k}}{2}. \end{array}\right. \end{array}$$

where $d^{k}$ is the distance between the left and right antenna, $d^{k}=\frac{\delta^{k}}{c}$, and *c* = 2.

In addition, the mutation operation generated by adding a Gauss perturbation on the swarm’s best position when the global best position of the swarm remains constant many times is added into the CBSO to prevent premature convergence. In this way, the algorithm can find a better solution in the solution space, reducing the possibility of falling into local optima. The perturbation equation is shown as follows:

(20) $$\begin{array}{c} G_{best}=G_{best}\left(1+normrnd\right). \end{array}$$

The parameter identification objective is to optimize the model parameters so as to minimize the error between the measured voltage $V_{0}\left(k\right)$ and the predictive voltage ${\hat{V}}_{o}\left(k\right)$. The optimization objective can be described as

(21) $$\begin{array}{c} minf\left(\cdot \right)=\sum_{k=1}^{M}{\left[V_{o}\left(k\right)-{\hat{V}}_{o}\left(k\right)\right]}^{2}, \end{array}$$

where f(·) is the fitness function, and *M* is the number of sampling points.

It should be noted that the boundary conditions of the algorithm influence parameter identification. Indeed, if the boundary conditions are set too small, then the algorithm may miss the optimal solution. If the boundary conditions are set too large, then the accuracy of the algorithm may be reduced.

**Figure 2:**
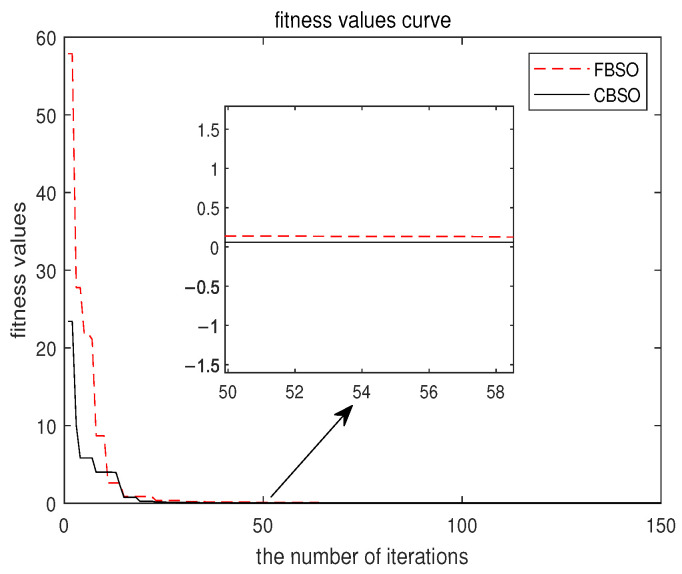
The fitness curves of the FBSO and CBSO.

**Figure 3:**
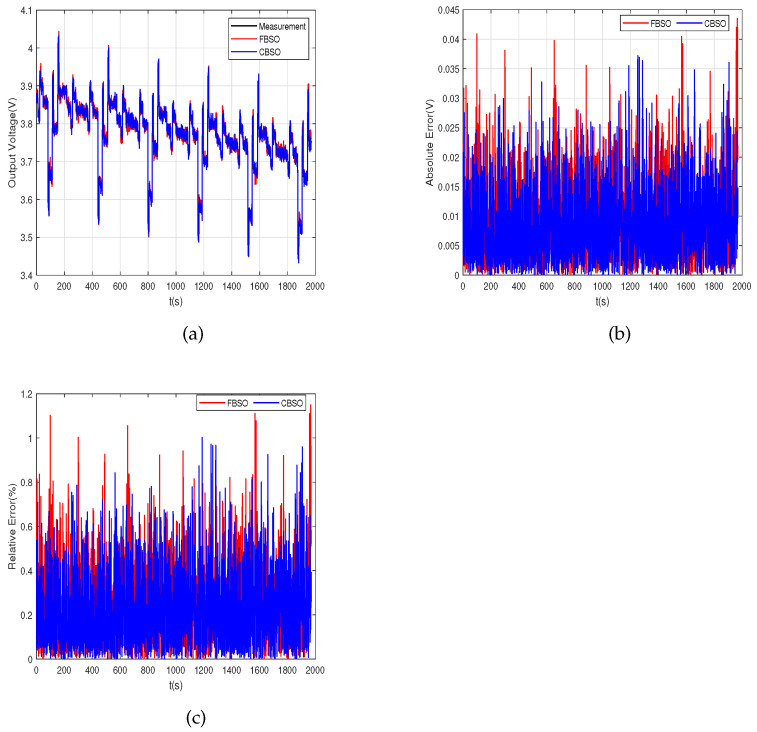
(**a**) Model accuracy verification with the FBSO and CBSO; (**b**) output terminals voltage absolute error; (**c**) output terminals’ voltage relative error.

To show the superiority of the novel CBSO algorithm, FBSO is chosen to compare with the proposed algorithm. The fitness value evolution curves of the two algorithms are shown in Figure 2. The red dotted line represents FBSO, and the black solid line represents CBSO. It can be derived that the convergence speed of CBSO is faster than of FBSO. The identification results of the OCV-SOC polynomial coefficients are listed in Table 1, and the results of the identification of the model parameters are listed in Table 2. The parameter identification results are shown in Figure 3. The red and blue lines stand for FBSO and CBSO, respectively. Furthermore, the two algorithms are compared with the measurement value represented by a black line. The absolute error (AE) and relative error (RE) of the two algorithms are shown in Figure 3b,c, respectively. It can be inferred that the maximum AE values of the two methods are 0.0435 V and 0.0372 V, and the maximum RE of the two methods are 1.15% and 1.00%, respectively. These numerical results show that CBSO not only has fast convergence speed but also has high precision.

Two metrics with RMSE and MAE were used to further evaluate algorithm accuracy. The RMSE and MAE of the two methods are summarized in Table 3. It can be derived that the metrics of CBSO are better than FBSO. The RMSE for the two methods is 11.7 mV and 10.8 mV, and the MAE is 9.3 mV and 8.6 mV, respectively. These results further show that the proposed method has higher accuracy, and they demonstrate the effectiveness of CBSO.

## 5. Conclusions

In this article, the CBSO was proposed to improve the accuracy of the battery model by employing the CO operator concept and mutation operation based on the BSO. The FOM was adopted for battery modeling based on the EIS. The CBSO updates the velocity formula based on the CO operator concept and introduces the mutation operation into the BSO algorithm, which can capture the particles’ memory and alleviate the issue of the algorithm falling into local optima. The maximum AE and RE of the CBSO are 0.0372 V and 1.00%, while the RMSE and MAE are 10.8 mV and 8.6 mV, respectively. The numerical experiment proves that the proposed method can significantly improve the LIBs model accuracy. The complex order will affect the efficiency and effectiveness of the identification of model parameters. In future work, we will address the impact of different parameter settings on identification accuracy.

## Figures and Tables

**Table 1:** The parameter identification results for the coefficients of OCV-SOC polynomial.

OCV-SOC	$a_{0}$	$a_{1}$	$a_{2}$	$a_{3}$	$a_{4}$
FBSO	3.8787	1.2571	2.3615	−0.8781	−0.0717
CBSO	3.8765	1.1046	0.8815	−0.2153	−2.0480

**Table 2:** The results of parameter identification with FBSO and CBSO.

	Resistors	Capacitors	Fractional Orders
FBSO	$R_{o}=0.0701$	$C_{1}=1059.9037$	$\alpha_{1}=0.9678$
	$R_{1}=0.8583$	$C_{2}=842.9402$	$\alpha_{2}=0.1351$
	$R_{2}=1.1919$	W=1041.3675	
CBSO	$R_{o}=0.0719$	$C_{1}=1133.0955$	$\alpha_{1}=0.9627$
	$R_{1}=0.7084$	$C_{2}=1038.1398$	$\alpha_{2}=0.5013$
	$R_{2}=1.0577$	W=1705.1727	

**Table 3:** The RMSE and MAE of the results of model parameter identification.

Metrics	RMSE (mV)	MAE (mV)
FBSO	11.7	9.3
CBSO	10.8	8.6

## Data Availability

Data is available online at https://web.calce.umd.edu/batteries/index.html, accessed on 1 February 2023.
